# Where I Belong: Identification Processes of Young Volunteers in Super-Diverse Cities

**DOI:** 10.1007/s11266-021-00404-z

**Published:** 2021-10-07

**Authors:** Marie Lehner, Astrid Mattes, Ilona van Breugel, Ursula Reeger, Peter Scholten

**Affiliations:** 1 Institute for Urban and Regional Research, Austrian Academy of Sciences, Postgasse 7/4/2, 1010 Vienna, Austria; 2 Department of Public Administration and Sociology, Erasmus University Rotterdam, Burg. Oudlaan 50, 3062 Rotterdam, Netherlands

**Keywords:** Politics of belonging, Young volunteers, Super-diversity, Urban spaces, Weak theory

## Abstract

In the context of super-diverse cities, scholars and policy makers are increasingly interested in the potential of volunteering to establish identification for newcomers and locals alike. In this paper, we address the question of how young volunteers in Rotterdam and Vienna negotiate belonging within their super-diverse surroundings. Our exploratory study builds on a cross-national research project in which we collected qualitative interview data from volunteering youth. We follow a weak-theory approach and conceptualise belonging as emotional, procedural, and relational. We trace identification processes of newcomers and locals in terms of belonging through volunteering in urban contexts of super-diversity. Our paper demonstrates that volunteering serves as a vehicle for feelings of belonging and inclusion for young volunteers, specifically addressing the urban super-diversity of Vienna and Rotterdam. Our research also indicates the partiality and temporality of volunteering as a source of belonging and the function of volunteering as a structure of inclusion, not necessarily enabling structural inclusion.

## Introduction

In the current age of migration (Castles & Miller, 2009), metropolitan areas are increasingly understood as spaces of super-diversity (Vertovec, 2007). In such complex social contexts, where categories of ‘immigrants’ and ‘natives’ or ‘us’ and ‘them’ hardly function, simplistic concepts of immigrant integration often fail to grasp the challenges of societal inclusion. Yet, some of the research trajectories that integration research has found beneficial are worth exploring, also in the specific constellation of super-diverse cities. One of these trajectories concerns volunteering, which has been identified as a facilitator that might serve as a catalyst in integration processes (see e.g. Wiertz, 2016; Davies, 2018 and Greenspan et al., 2018). Seeing that many studies confirm the beneficial potential of volunteering, this hope is not unsubstantiated and has made its way into urban public policies. The UK capital of London, to name but one example, has launched an integration strategy building on voluntary engagement of immigrants (https://www.unv.org/swvr/volunteering-tool-social-integration-cities). Similar efforts are made in Vienna (https://www.refugees.wien/initiativen-fuer-integration/) and Rotterdam (https://rotterdammakeithappen.nl/showcases/rotterdammers-helpen-elkaar/), the cities we observe in this study. However, there is little knowledge of the social processes that volunteering might trigger, especially in the light of super-diversity, which considers the dynamic interplay between multiple categories of difference.

In this paper, we examine the identification processes that volunteering triggers among young people in the particular context of super-diverse cities. Rather than applying the concept of integration, which differentiates between ‘natives’ and ‘immigrants’, we build on ideas of belonging to grasp complex realities of identification processes in super-diverse environments. By focusing on young people who are undergoing formative phases in their personal development and whose identification processes are largely fluid and ongoing (Habib & Ward, 2019), we add an additional layer of complexity to our analysis of volunteering and belonging.

For this exploratory multiple-case study, we build on qualitative interview data from interviews with young volunteers in Vienna and Rotterdam, which were conducted as part of a larger European research project. Informed by research from the field of urban studies, we draw on a ‘weak-theory approach’, which allows for the emergence of new concepts within the research process. Thereby, we acknowledge researchers’ criticism of the integrative potential of volunteering that emphasizes the pre-determined findings of many studies in this field (McLean et al., 2002). Accordingly, we move beyond utility-oriented approaches of volunteering as a tool for integrating immigrants.

The paper proceeds as follows: First, we map the field of theoretical discussions of volunteering, belonging, and super-diverse cities. This is followed by the introduction of our analytical framework and the description of our research design and data material. Then we discuss the empirical findings and theoretical implications, and end with a short, conclusive summary.

## Volunteering and Belonging in the Super-Diverse City: Mapping the Field

Volunteering can enhance civic participation and is commonly understood as fostering integration (see e.g. Handy & Greenspan, 2009; Guo, 2014; Baert and Vujic, 2016; Greenspan et al., 2018). Many beneficial aspects are ascribed to volunteering, including social-capital building (Putnam, 2000), informal learning (Guo, 2014), and empowering volunteers towards active citizenship (see Verthongen et al. 2017). However, these beneficial aspects of volunteering are primarily understood from a socio-economic perspective and are mostly used as measurement of (un)successful integration (see Wierts 2016). In these cases, integration is often understood as a desired degree of participation in or adaptation to the host society, in which volunteering is considered to mirror a level of civic engagement (Ishizawa, 2015). Both the role of the individual experience of volunteers and that of mutual cultural exchange between different groups forming the super-diverse society are mostly overlooked. In one of the studies that does focus explicitly on the immigrant volunteering experience, Handy and Greenspan (2009) find that immigrant volunteers are searching for social connections and wish to improve their language, culture, and practicalities of daily life, rather than access the labour market. Tomlinson and Erel 2005 (in Vaccheli and Peyrefitte 2018) argue for considering volunteering not only as a tool towards integration, but also as a means of shaping and improving society as a whole. In this paper, we thus do not speak of integration but rather look at the role of volunteering in the identification processes of young people (both immigrant and non-immigrant) in a super-diverse urban context.

In this vein, von Essen (2016; 2020), Grönlund (2011), and Wuthnow (1991), among others, write about volunteering as promoting a sense of belonging. “The crucial function of referring to engagement is to make volunteering into actions that are intimately connected to themselves and to disclose who they are as persons” (von Essen, 2020: 251). Grönlund argues for a research approach combining identifications, values, and volunteering. The author finds that volunteering was used “as a way to be the person they felt they were or wanted to be morally” (2011: 568), stretching beyond their role in volunteering organisations. Young volunteers form a particularly interesting group against which to study this process of identification and belonging, given the formative phase the youngsters go through (Taylor-Collins et al., 2019). As Rosenthal and Bogner point out, questions of belonging are not equally relevant in every phase of life. However, they are specifically important at biographical turning points and in times of change (Rosenthal & Bogner, 2009; Grönlund, 2011; Davies, 2018; Verthongen 2017; Habib & Ward, 2019). This study thus explicitly focuses on the experiences of young volunteers with and without biographical links to migration, to explore identification processes and belonging in a super-diverse urban context.

Both the concept of belonging and the idea of super-diversity are increasingly applied in migration studies, as they are suited to grasp contemporary urban settings and the identification processes of their inhabitants. Super-diversity is an overarching term for “drawing attention to new social complexities, […] new social patterns, forms and identities arising from migration” (Vertovec, 2019: 125). The idea of super-diversity emphasizes the multi-dimensionality of diversity, stretching beyond mere ethnicity or nationality and including other forms of diversity, such as age, gender, level of education, and lifestyle (see e.g. Vertovec, 2007, Crul et. al. 2013). This indicates the degree of diversity within the migration population as well as the increasing diversity of societies overall. Unlike the rather essentialist ideas of ‘migrants’ and ‘natives’, super-diversity describes social realities determined by more complex and fluid patterns of belonging that not only take newcomers into consideration but also considers the situatedness of diverse individuals in general.

The concept furthermore draws attention to diversity as a characteristic of (contemporary) societies in general and urban contexts in particular (Wessendorf, 2014 on commonplace diversity). Studies have consistently shown that marginalised groups particularly identify more easily with the local level than with the idea of belonging to a nation state (Crul & Schneider, 2010). In fact, identification and feelings of belonging on the local level of the city have been discussed as alternatives to collective identity concepts on the national level, which are often problematic, also in conceptual terms (Bauder, 2016; Koefoed & Simonsen, 2011; Simsek-Caglar and Schiller 2018). Therefore, super-diverse urban contexts are an especially interesting field for the investigation of the relation between volunteering and belonging.

Building on constructivist literature, we use the concept of belonging, which is essentially a procedural category. We avoid the concept of identity, which is often seen as a “possessive property of individuals” (Anthias, 2018). As Floya Anthias states, “belonging and identity can be seen as part of the same ‘family’ of concepts and, while both are used politically in similar ways, belonging enables a greater engagement with place and location and the structural and contextual facets of social life” (2018: 137). In following Montserrat Guiberneau, we perceive belonging as an individual’s deliberate claim to membership to socially constructed collectivities (2013). We thus understand identification as the agentive, creative process leading to belonging. Whereas literature on volunteering often simply refers to identity without discussing the details of the concept, we consider it useful to incorporate the related but more refined concept of belonging, with identification as its procedural element.

Rooted in the theory on intersectionality (Crowley, 1999; Anthias, 2006; Yuval-Davis, 2006, 2011; Guibernau, 2013), the literature on belonging and politics of belonging is increasingly being applied in other fields concerned with societal membership. Scholars of volunteering who have adopted the belonging approach have drawn attention to the individual identification processes taking place among refugees along the pathways from voluntary work to paid employment (Tomlinson, 2010) and to volunteering as a resource for identification processes in times of crisis (Carlton, 2015). As pointed out by Hustinx et al. (2010), volunteering is widely seen as contributing to social integration and inclusion, but the findings of Dallimore et al., among others, illustrate that this “is just one side of a dichotomous picture that also sees volunteering as a vehicle for division and exclusion” (2019: 19). Belonging takes into account the fluidity and multi-dimensionality of identification processes as well as the constraints placed on young volunteers through structures of exclusion.

While volunteering can be viewed as a series of processes of identification with organisations, goals, and fellow volunteers, it is important to note how this relates to other (particularly spatial) identification processes, to situate the process of belonging of the volunteer in the city. Dallimore et al. (2018) have investigated this from an ethnographic perspective. They enquire after the relevance of local embeddedness for volunteering activities. Their findings illustrate the relevance of localities for volunteers and volunteering, but they also encounter significant variation regarding the spatial identification of volunteers, despite comparing similar cases of North-Welsh small towns. Local specifics and individual biographies are both decisive for feelings of belonging and consequently for the inclusionary effects of volunteering. Similarly, Dahlvik et al. (2017) identify strong divergences in terms of neighbourhood initiatives and belonging in the super-diverse urban areas of Amsterdam and Vienna.

## Analytical Framework

Belonging, which is understood to be constructed continuously through individual and collective processes of identification, remains a complex concept that escapes rigorous theorisation. As suggested by Sarah Wright (2015), we treat the ambiguity of belonging as a conceptual strength in the sense of a weak-theory approach that allows for an explorative element. In contrast to strong or grand theories, weak theories do not claim completeness, but rather approach phenomena openly (Fotion, 2014). They wish to theorise as “ontologies immersed in the middle of things” (Stewart, 2008: 77). Thereby, weak theory “observes, interprets, and yields to emerging knowledge”, rather than structure what we already know (Gibson-Graham, 2014: 149). It is the theoretical equivalent of explorative empirical research, related to ethnographic and thick-description approaches as introduced by Clifford Geertz (1973) and others. This attempt towards a performative ontology postulates that we should accept that our representation of the world contributes to our enactment of that world (Gibson-Graham, 2014: 149). This approach contrasts strong theories with broader explanatory aspirations that aim to structure phenomena in a seemingly predictable manner.

As Wright (2015) points out, a weak-theory approach to belonging has a series of conceptual consequences. Firstly, such an approach puts emotions at the centre of scholarly attention, thereby exploring the *emotionality of belonging*. This is often mentioned but hardly ever researched explicitly. Secondly, as pointed out in most works on the concept, and related to the importance of emotions and affect, belonging, as a weak-theory concept, must be approached through its *procedural character*. “Attention to practice, then, is very useful in highlighting the way that belonging is continually (re)made and (re)constituted, and how it is performed in messy, negotiated and material ways” (Wright, 2015: 400). Thirdly, a weak-theory approach to belonging calls for a *relational perspective*, which is often overlooked in other studies. Sarah Wright borrows from indigenous concepts and ethnographic studies in approaching belonging as the relational configuration of entities that come together. As she sees it, “[t]hings (or people or places) do not pre-exist, in static ways—their belongings are made through their coming together. Food, organisms, trees, music, markets, hair and dance all actively co-constitute belonging” (Wright, 2015: 403). For us, the inclusion of spaces, places, and artefacts is particularly suitable as we investigate belonging in super-diverse urban contexts. We use these insights that understand belonging as an emotion, a process, and a relation to structure our findings on identification processes of young volunteers.

For our analysis, we combine this approach from the literature on belonging as a weak-theory concept with works from urban studies, migration studies, and human geography. We particularly look at the importance of *urban localities* for emotions, processes, and relations of belonging. Here, we also build on Antonsich’s idea of place belongingness, which refers to a personal, intimate feeling of being ‘at home’ in a place (2010). We investigate *ideas of the city* to understand how urban super-diversity as an abstractum relates to emotions, processes, and relations of belonging. As Edward Soja’s concept of urban imaginaries (2000) suggests, we expect cognitive conceptions of an urban social reality to be relevant for identification processes and feelings of belonging. Suzanne M. Hall speaks of the ‘symbolic city’ when referring to abstract notions of the city as a whole (2015), and Myrte Hoekstra (2018) suggests that ideas of the city are meta-narratives that also pertain to the understanding of the place of migrants in the urban community. In her essay on belonging, space, and marginalised young people, Kitty te Riele (2018) points to relational aspects of spaces and the inter-personal connections of *individuals and groups*. Individuals, groups, and group dynamics also form the starting point for Steven Vertovec’s thinking about super-diversity (2007). Together with urban localities and ideas about the city, we include individuals and groups within the city as a third dimension for the analysis of belonging, which we perceive as *emotional, procedural, and relational*.

## Research Design, Methodology, and Data

We research the role of volunteering for belonging in super-diverse urban contexts by asking ‘How do young volunteers in super-diverse contexts negotiate belonging, understood as an emotion, a process, and a relation?’ We investigate the specifics in urban young people’s negotiation of belonging that relate to their volunteering activities. For this purpose, we draw on qualitative interview data of young people who had engaged in volunteering during the study, as they form a particularly interesting group for researching the relation of volunteering and belonging, given the formative phases that youngsters go through (c.f. Taylor-Collins et al., 2019). For the purpose of our study, volunteering was defined as a continuing activity “carried out throughout an association or organisation willingly and without being forced or paid to do it” (Flarer et al., 2021: 6).

We focus on two urban contexts of super-diversity: Rotterdam and Vienna. Both cities have a rich tradition of migration and the inclusion of newcomers, which resulted in an ethnically, religiously, and lingually heterogeneous urban population. These cities are and have been attractive for newcomers for many decades and are thus important arrival cities in the respective national contexts. Following many decades of immigration, Rotterdam has evolved into a super-diverse city, where more than half of the population has either a first- or a second-generation migration background, involving more than 180 different nationalities. We see a similar picture in Vienna. Super-diversity, however, concerns more than passports, as the concept takes into consideration the presence of diversity in various forms, such as religious affiliation, gender identification. Both cities are characterised by these multidimensional complexities.

Our explorative multiple-case study (Stake, 2013) involves locals and newcomers who are beginning to engage in volunteering in these cities. We are aware of the limitations of this specific research design. A series of challenges occur when subsuming data collected in different contexts, even though multiple case studies do allow for richer data collection. Rather than comparing results from Rotterdam and Vienna, we are interested in the particularities of belonging influenced by volunteering. Nonetheless, we will reflect on the similarities and differences in our empirical findings for these two cities.

In total, we conducted 20 qualitative interviews over 14 months (see Appendix) and interviewed each volunteer twice.^1^ Our sample includes five males and five females between the ages of 18 and 27, residing in Vienna or Rotterdam. For half of them, migration played a significant biographical role. This composition of a sample consisting of locals and newcomers results from the overall project, VOLPOWER, of which this research is a part. VOLPOWER examined how youth volunteering in sports and arts activities can serve as a mechanism enhancing social cohesion among diverse youths across Europe (see www.volpower.eu). This article presents a data sub-set that focuses on the belonging of young locals and newcomers who start to volunteer in a super-diverse city. A strict differentiation between locals and newcomers was not the focus of our sub-study, as we approach migration as a highly common, even characteristic phenomenon of the super-diverse city.

We interviewed each volunteer twice, using longitudinal qualitative interview techniques. The second wave of interviews took place during the beginning of the COVID-19 pandemic in the spring of 2020 (March–May). Therefore, the second wave of interviews was largely conducted online or via telephone. It must be assumed that this methodological change, as well as the extraordinary circumstances during local lockdowns, have affected the course of this second wave of interviews, although the extent of this effect cannot be assessed. Having asked our interview partners to reflect upon the past year, in which they all have started their volunteering engagements, thereby addressing a longer period before the outbreak of the pandemic, we hope to have contained the impact of the current health crisis on our data collection. Furthermore, as our interviewees were part of a larger study, the researchers and the interviewees already knew each other and a certain level of familiarity existed. We assume that this stable relationship enhances the quality of our data, although we are aware of the limitations imposed on social scientific research during a pandemic (Bania & Dubey, 2020).

The volunteers had hosting, organisational, and coaching roles in different organisations across Vienna and Rotterdam. In Vienna, volunteers were involved in football clubs and a climbing organisation. In Rotterdam, the interviewees volunteered at a dance community and at an organisation that works with Eritrean refugees, offering consultation as well as sports and cultural activities. The interviewees started their engagement in these organisations at the beginning of the research period and volunteered at least until the second round of interviews was conducted.

The qualitative guideline questionnaires covered a broad spectrum of themes and were structured according to the concepts of ‘volunteering’, ‘belonging’, ‘empowerment’, and ‘interpersonal relations’. In the first wave, we asked about expectations of volunteering, while interviewees narrated their collected experiences in the second wave. When discussing belonging, we enquired after individual feelings of belonging via the concepts of inclusion and exclusion and in relation to the current place of living (see discussion above, as well as Youkhana, 2015), using the same set of questions in both waves. We for example asked the interviewees about places in which they felt, especially comfortable or uncomfortable and enquired about factors contributing to their sense of inclusion or exclusion in Rotterdam and Vienna, respectively. We addressed empowerment and its potential restraints through questions on self-determination and decision-making processes (see Alsop & Heinsohn, 2005) similarly in both rounds of interviews. Regarding interpersonal relations, we used a social contact map (see Greene & Hogan, 2005), where interviewees mapped their most important contacts with family, friends, colleagues, and fellow volunteers.

Interviews were recorded, transcribed, and analysed in three steps. First, we coded and analysed each interview individually using thematic analysis (Nowell et al., 2017). In a second step, we used the analytical framework presented in Table 1 to structure the findings of our thematic analysis. Where applicable, we assigned the thematically coded passages deductively to the characteristics of the super-diverse city (*ideas*, *places,* and *people*). Thirdly, we discussed the role of volunteering for emotions, processes, and relations for each of these aspects in the super-diverse city. The data examples in Table 1 demonstrate how we proceeded in our analysis.

**Table 1:** Analytical framework and exemplary codes (own conceptualization)

			Belonging as (Wright, 2015)		
			An emotion affects feelings	A process becoming practice change	A relation (‘more than human’) places artefacts
Super-diverse city	Urban localities (Antonsich, 2010)	e.g. St. Stephen’s cathedral (AT 8)	A feeling of inclusion	Taking walks in the city	My Vienna emerges
	Ideas of the city (Hall, 2015; Hoekstra, 2018; Soja, 2000)	e.g. Vienna is very liberal (AT 4)	Feeling alike	Easily moving into bubbles	Choosing people
	Diverse individuals and groups in the city (te Riele, 2018)	e.g. Becoming part of the football coach group (AT1)	Feeling strange/not yet familiar	Getting to know each other over breakfast	Presenting a new group as team

## Volunteering and Belonging: Insights From Empirical Research

Our study was guided by the research question regarding young urban volunteers’ negotiation of belonging. We subsequently present findings from the empirical analysis along the axis of elements of super-diverse cities that we have identified (urban localities, ideas of the city, diverse individuals and groups), highlighting elements from our analytical framework in small caps and *italics*. This is followed by a discussion of consistencies and fractures in our results that demonstrate other relevant aspects, such as the particularities of urban contexts and the impact of migration biographies.

### Volunteering Sites as Significant Urban Localities

Significantly, young volunteers in both Vienna and Rotterdam do consider volunteering a part of their broader sense of belonging, as it enables them to connect to certain *places*. Interviewees describe sites of volunteering as *urban localities* of emergent positive feelings of belonging.

Conversely, physical presence and volunteering activities in the city also strengthen the volunteers’ sense of belonging. This process relates to *people* as well as to *places.* It concerns the building of relations and memories in the city, which add to their sense of belonging. As remarked by one of the Viennese volunteers:

… [L]ike where we had a match or where we trained or where we met, and that now reminds me that I have a lot of good friends here that I can meet or talk to, and this city is all of us together. That gives me a good feeling when I think that I also belong to it... (Viennese volunteer 2).

Furthermore, feelings of belonging in the volunteering context connect to the responsibilities that volunteers take at their specific volunteering organisations:

“I think there was a development in the direction that I feel more included and more involved. In [volunteering organisation, VO], it was very clear that everyone just took care of something. That means we do everything together and everybody gets a task and not just the ones who are the loudest. … So I made new friends and you feel included and comfortable when you are in a room where you can look at people as friends” (Viennese volunteer 1).

The fellow volunteers became good friends and at urban sites of volunteering, place-belongingness (Antonsich, 2010) merges with individual politics of belonging in references to social collectivities (Yuval-Davis, 2006). However, whereas volunteering and the *people* and *places* of volunteering play an important role as exemplary places of urban diversity and inclusion, these connections and ideas are not unique to the volunteering context.

For the volunteers, belonging can emerge at different locations and levels, and it is strongly tied to feeling at home with people. Belonging is thus more social than spatial. Volunteering organisations are an important place where such connections are developed, but again, they are not unique. In this sense, the spatial dimension of belonging becomes evident in their volunteering organisation as a place that also reinforces the volunteers’ connection to the city. As one of the Rotterdam volunteers describes:

It is about knowing people ... When you arrive at [VO], it is just nice to see the faces of people ... It is just nice to see people and talk to them. That is what feeling at home can be to me too … I now really consider Rotterdam my home, because of two things. Because my friends live there and because I come to the [VO] every day. At least five times a week. That is why I consider Rotterdam my city and why I feel at home in Rotterdam (Rotterdam volunteer 2).

### Volunteers and Their Ideas of the City

In Rotterdam, the volunteers particularly refer to the specific attitude of openness and the self-evident notion of diversity (Wessendorf, 2014: ‘commonplace diversity’) in the city as well as at their volunteering organisations. To them, diversity signifies encountering diverse people and groups, which happens at their volunteering organisation. They consider this a self-evident part of their volunteering experience. Two volunteers also refer to personal growth through volunteering. This involves learning more about different types of diversity (including sexuality, femininity and masculinity, etc.), through encountering diversity in their volunteering contexts and using this as a mirror for their own positions and views, or even advocating diversity.

“You encounter it a lot [diversity], also in terms of sexuality etc. The nice thing about [VO] is that it does not matter. … You all come for the same goal, you are all part of the community, regardless of your background. … It [volunteering] stimulates me in advocating diversity“ (Rotterdam volunteer 3).

The volunteers consider their volunteering organisations as exemplary places of urban diversity. One of the Rotterdam volunteers remarks:

“Rotterdam is a multicultural city. [VO] really mirrors that. … Every time you enter [VO] you encounter different cultures. It is not something remarkable to me. It is normal, that just happens here” (Rotterdam volunteer 2).

Here, we see references both to the site of volunteering as a *place* of belonging and to Rotterdam as a certain urban imaginary (Hoekstra, 2018). The quotes illustrate that volunteers assign certain characteristics to their cities, which are experienced and strengthened through their volunteering experience. Rotterdam is perceived as multicultural and super-diverse in a very nonchalant way. It is a city to which the volunteers closely relate. Vienna is likewise perceived as a liberal, multicultural city, and interviewees describe their volunteering work as an opportunity to shape their surroundings in accordance with their *ideas of the city*, whereby their *places* of belonging emerge relationally.

That’s my city and that’s one of the main reasons why I think I wanted to get involved in social life, which is not because I have such great morals now, but because that’s my Vienna and I do something there [about the situation of refugees] (Viennese volunteer 8).

In this quote, the interviewee also describes a feeling of being capable to make an impact through volunteering activities that is related to the feeling of belonging to the city.

### Volunteering and Belonging of Diverse Groups and Individuals

Rather than being forced to deal with all contemporaries, volunteers describe a process of choosing ‘bubbles’ that they are willing to be part of, of which the volunteering situation is one. This element of choice and companionship by choice was an important process described by volunteers. The volunteers remark that they feel free to make their own choices, and that volunteering strengthens them in this regard. They consider their volunteering choice as their own deliberate decision, an outcome and further extension of their freedom.

The procedural character of this urban like-mindedness also leads to fluid but somewhat unstable feelings of belonging in relation to different groups in the city. As one of the Viennese volunteers describes:

“I don't know, it’s just, in Vienna it’s easy to move in bubbles. Well, I almost only meet people who study or are at least half left-wing or very left-wing^2^ [laughs], but I never meet the others” (Viennese volunteer 4).

However, the selective and temporal elements of getting together in the volunteering context are mentioned too. Whereas belonging to their organisation and to the city forms an important part of their lives for most volunteers, this is clearly *bound in time and space* for one of the volunteers:

“Well, it’s a community, definitely, but it’s what I would call a temporary community. So again and again, suddenly, full of community and then, again, fully away” (Viennese volunteer 5)*.*

Furthermore, feelings of belonging, whether temporal or not, exist alongside feelings of alienation elsewhere, for example contrasting the open and welcoming attitude within the volunteering organisation to a more hostile or exclusive environment in the wider city:

“Otherwise I feel I don’t belong anywhere, except when I really feel good with some people. And if I feel good in Vienna, it’s because of the people in [VO], work colleagues, study colleagues, roommates … ” (Viennese volunteer 7).

Experiences of belonging can also transcend the organisation or city. One of the Rotterdam volunteers for example refers to the open attitude that is characteristic of her volunteering organisation and the international dance community alike. These communities are characterised by diversity, yet she also specifically refers to feeling at home with a specific attitude, which she calls “belonging to a social equality community”:

“Everyone can say what they want. Everyone listens to you. It does not matter who you are. If you have something to say, we will listen, whether we agree or not. You are free there” (Rotterdam volunteer 2).

Her connection through volunteering thus extends beyond the specific urban context. She feels part of something bigger within the international dance community but also observes a broader attitude that is characteristic of her generation. Another Rotterdam volunteer refers to specific characteristics of volunteering itself, beyond her organisation. Rather, this interviewee describes identification at the individual level, based on the desire to help people:

“Well, I happen to volunteer with Eritrean newcomers now, as I speak their language, but to me it is about being a person who is open and willing to help others voluntarily. It can be elderly people or non-native speakers, whoever. It just happens to be Eritreans now but it could have been anyone. It is about being a volunteer” (Rotterdam volunteer 3).

### Discussing Consistencies and Fractures in Our Results

Interviewees show awareness of structural aspects in processes of exclusion in the city. They describe volunteering as a relational, inclusive experience alongside other inclusive *urban spaces* and *group experiences* in their daily lives. However, this partial and temporary inclusion does not overcome structures of exclusion. Contradictory feelings of inclusion and exclusion, belonging and alienation, occur simultaneously in the urban setting. As one of the Viennese volunteers describes:

I have always said that I loved Vienna, but I merely like it now, because I was disappointed by Vienna, by the political situation [xenophobia]. Yes, but that’s just the way it is, what should I do (Viennese volunteer 7).

Many volunteers contrast their experiences of inclusion in the city and in their volunteering organisation with feelings of exclusion elsewhere. One of the Rotterdam volunteers for example states really feeling at home in urban settings, but not in the Netherlands as a whole. She does not feel at home in rural areas, feeling more conspicuous there than in the city. While this is partially overcome through the urban volunteering experiences of inclusion, this does not change her not feeling at home in the Netherlands. Other volunteers also reflect on the limitations to their senses of inclusion:

If an enemy image is created against a certain group and is then enforced, this leads to the fact that this group also leaves society and society also orients itself against this group and then everything happens except integration and inclusion (Viennese volunteer 1).

Especially for those volunteers who have a migration history or an indeterminate legal status, structural exclusion is a pressing issue. Here, we see the most striking difference between locals and newcomers: Recent migrants have a strong awareness of the fragility of their inclusion. Volunteering might support the identification with local *places, spaces, and ideas*, but the insecurity prevails. Although the concept of urban super-diversity treats migration and migration-driven diversity as urban normality (Vertovec, 2007), and newcomers among the volunteers state their feelings of belonging as emergent in the urban volunteering context, the factual difference created by legal categories overshadows these feelings and renders them deficient. We clearly see that volunteering provides a structure of inclusion, but not of structural inclusion. Another fracture that we observe in our data concerns differences between Rotterdam and Vienna. The two cities differ in the perception of volunteers, since diversity is described as very commonplace and self-evident in Rotterdam, while differences among the Viennese populations are discussed as positive yet challenging. This might relate to differing dominant migration narratives and models of integration in Austria and the Netherlands (Bertossi, 2011; Favell, 1998; Joppke, 2007). While both national contexts have seen immigration over the past decades, their models or philosophies of integration and citizenship differ. This is somewhat surprising as our results point to a rather trite theoretical discussion for differences in the inclusion of immigrants, namely national models. Migration research has largely rejected this argumentation, particularly on the local level (Wimmer & Glick Schiller, 2003). Nonetheless, our empirical results indicate the relevance of national narratives in shaping the perceptions of our volunteers and influencing the frame in which they negotiate their belonging to the super-diverse cities in which they live.

## Conclusion

In this paper, we have analysed the identification processes triggered amongst local and newcomer youth volunteers in the context of two super-diverse cities, Vienna and Rotterdam. Our results demonstrate the potential of volunteering to strengthen the identification of young urban people with their city and the people they meet there, but equally reveal its limits. As often occurs when dealing with aspects of inclusion, the importance of structural inclusion—understood as a secured livelihood in the current place of living—prevails over all other forms of inclusion (Ager & Strang, 2008). Nonetheless, where structural inclusion is not (yet) given, our exploratory study shows that volunteering can strengthen feelings of belonging at a smaller scale. Along with many other emotions, processes, and relations in the lives of young people, volunteering contributes to identification with and in the super-diverse city.

In our study, volunteering facilitates specific aspects of identification. We find freedom of choice, as is the case in voluntary engagement, to be an important aspect of identification processes (Handy & Greenspan, 2009; Wilson, 2012). While this choice is not limitless, it is deliberate. In this sense, the feeling of being at home somewhere has an element of choice per se. Our analytical framework proved suitable, as we found rich evidence of the emergence of such feelings of belonging in relation to people, places, and certain ideas. Our explorative study furthermore displays the high interconnectedness of these elements and the ways in which they reinforce each other. Following the insights of our analysis, we move beyond the focus on volunteer engagement and identification at the individual level (von Essen, 2020). We propose an understanding of belonging as the deliberate procedural, relational, and emotional choice to accept and proclaim places, ideas, and people as part of one’s own self-perception. The latter however remains fluid and is influenced by changes both in the decisions and in the circumstances of individuals. In this study, volunteering is shown to provide places and ideas which and people whom an individual might choose to identify with, as well as an opportunity to make these choices. By demonstrating these processes, we hope to contribute an applicable framework for the structural research of belonging in super-diverse urban contexts. This approach to belonging and the ability to provide for identification processes is important for the field of volunteering. Not only is it relevant for the recruitment of volunteers, but it also allows for new concepts in the interaction with volunteers. Addressing the elements of deliberate choice, feeling at home, and parallels of inclusion and exclusion might help to support young volunteers in super-diverse urban settings in their negotiation of belonging, thereby strengthening their identification with their voluntary engagement. As literature underlines the importance of youth as a formative phase for identification processes, the insights from our explorative study on young volunteers should be further researched, using a larger sample and a comparative angle. We found that young people are very conscious about their negotiations of belonging, making them a particularly suitable group to study this concept.

In our study, the positive relation between volunteering and spatial identification is equally observable among locals and newcomers, in Rotterdam as well as in Vienna. While spatial aspects are emphasised in the literature and certainly should not be overlooked, social aspects of belonging shape the spatial perception of our interviewees (Anthias, 2018). Urban localities are related to feelings of belonging, primarily as sites of social interaction. As we were interested in the context of volunteering, which is by nature a social one, there might be more to discover about the spatial aspects of belonging. However, our findings closely tie spatial aspects of belonging to sites of friendship and group interaction. Such spatial aspects of belonging particularly seem to matter in the super-diverse contexts of Vienna and Rotterdam, where group categorizations and demarcations are complex (Vertovec, 2007 and Wessendorf, 2014). Our exploratory study suggests that urban volunteering could provide opportunities for identification or a 'structure for inclusion ' in the context of social complexity, often at the very basic level of friendship and interaction. However, we also found that this does not necessarily bring about structural inclusion. Discourses on migrant categorisations, differentiations between 'us' and 'them', often taking place on other levels such as the national, also have a clear pervasive effect on urban interactions.

Clearly, the exploratory research design of this study has multiple limitations. The challenges of qualitative research during a pandemic, as well as the somewhat different settings in Vienna and Rotterdam, impose certain restrictions. By studying volunteering and its relation to belonging in two super-diverse urban contexts, we approached a generally under-researched context in the study of volunteering. Future research could explore additional super-diverse contexts more systematically to enable comparison. Furthermore, it would be interesting to take the indications from our explorative study as a starting point for further studies to investigate the interplay of national models, philosophies, and narratives with local feelings of belonging. Here, potential rivalries of narrations in the contexts of volunteering, urban living, and nation-state structures could be investigated to enhance our understanding of barriers on the way to more inclusive societies. Following the insights of our study, we think that the analytical framework we proposed is a useful tool for the further analysis of these different aspects of youth volunteering and belonging in super-diverse cities.

## Appendix

Table 2.

**Table 2:** Overview interviewees

Interview no	Gender	Age	Place	Date
Rotterdam 1_1	Female	25	Rotterdam	May 28, 2019
Rotterdam 1_2			By phone	April 3, 2020
Rotterdam 2_1	Male	23	Delft	May 30, 2019
Rotterdam 2_2			By phone	April 1, 2020
Rotterdam 3_1	Female	27	Rotterdam	June 3, 2019
Rotterdam 3_2			By phone	March 29, 2020
Rotterdam 4_1	Female	20	Partly conducted in Zagreb during project training	June 14, 2019
Rotterdam 4_2			By phone	May 30, 2020
Vienna 1_1	Male	19	Vienna	May 22, 2019
Vienna 1_2			Skype	April 2, 2020
Vienna 2_1	Female	25	Vienna	May 17, 2019
Vienna 2_2			Skype	April 9, 2020
Vienna 4_1	Male	27	Vienna	May 23, 2019
Vienna 4_2			Skype	April 2, 2020
Vienna 5_1	Female	19	Vienna	May 27, 2020
Vienna 5_2			Skype	March 31, 2020
Vienna 7_1	Male	27	Vienna	May 31, 2019
Vienna 7_2			Skype	April 1, 2020
Vienna 8_1	Male	27	Vienna	May 5, 2019
Vienna 8_2			Skype	April 3, 2020

Please note: certain interviews were conducted online via Skype or over the telephone, as legal regulations due to the COVID-19 pandemic prevented face-to-face meetings in the spring of 2020

## Footnotes

1 In the Dutch case, some participants quit their volunteering engagement before the second wave of interviews. For this paper, we include only those participants that continued their volunteering at least up to the second wave of interviews.

2 Referring to the political spectrum.

## References

[CR1] Ager A, Strang A (2008). Understanding integration: A conceptual framework. Journal of Refugee Studies.

[CR2] Alsop, R. & Heinsohn, N. (2005). Measuring empowerment in practice: Structuring analysis and framing indicators. Policy research working papers. The World Bank. 10.1596/1813-9450-3510.

[CR3] Anthias F, Yuval-Davis N, Kannabiran K, Vieten U (2006). Belonging and identity are words overused and under-theorised in the context of population movements and translocation. The situated politics of belonging.

[CR4] Anthias F, von Davis K, Ghorashi H, Smets P (2018). Identity and belonging: Conceptualizations and reframings through a translocational lens. Contested belonging spaces, practices, biographies.

[CR5] Antonsich M (2010). Searching for belonging—an analytical framework: Searching for belonging. Geography Compass.

[CR6] Bania, J., & Dubey, R. (2020). The Covid-19 pandemic and social science (Qualitative) research: An epistemological analysis. Guni Network. Accessed at January 15, 2021 http://www.guninetwork.org/report/covid-19-pandemic-and-social-science-qualitative-research-epistemological-analysis.

[CR7] Bauder H (2016). Possibilities of urban belonging. Antipode.

[CR8] Bertossi C (2011). National models of integration in Europe A comparative and critical analysis. American Behavioral Scientist.

[CR9] Carlton S (2015). Connecting, belonging: volunteering, wellbeing and leadership among refugee youth. International Journal of Disaster Risk Reduction.

[CR10] Castles S, Miller MJ (2009). The age of migration: International population movements in the modern World.

[CR11] Crowley J, Favell A, Geddes A (1999). The politics of belonging: some theoretical considerations. The politics of belonging: Migrants and minorities in contemporary Europe.

[CR12] Crul M, Schneider J (2010). Comparative integration context theory: Participation and belonging in new diverse European cities. Ethnic and Racial Studies.

[CR13] Crul M, Schneider J, Lelie F (2013). Super-diversity. A new perspective on integration.

[CR14] Dahlvik J, Franz Y, Hoekstra M, Kohlbacher J (2017). Neighbourhood initiatives and belonging in super-diverse neighbourhoods in Amsterdam and Vienna: A comparative and applied-oriented analysis of neighbourhood-related policies.

[CR15] Dallimore DJ, Davis H, Eichsteller M, Mann R (2018). Place, belonging and the determinants of volunteering. Voluntary Sector Review.

[CR16] Davies, J. (2018). Young people & volunteering. attitudes and experiences. PhD Thesis. Glasgow: University of Strathclyde.

[CR17] Favell A (1998). Philosophies of integration: Immigration and the idea of citizenship in France and Britain.

[CR18] Flarer, H., Carla, A., Lehner, M., Mattes, A., Reeger, U. (2021). The effect of volunteering on empowerment and inclusion among EU and third-country national youth: An in-depth mixed-methods research. Accessed on July 07 2021, http://www.eurac.edu/en/research/projects/Documents/VOLPOWER%20WP2%20Final%20report.pdf.

[CR19] Fotion, N. (2014). Theory vs. anti-theory in ethics: A misconceived conflict. Oxford University Press.

[CR20] Geertz, C. (1973). Thick description: toward an interpretive theory of culture. In *The interpretation of cultures: Selected essays*. pp. 3–30. New York: Basic Books.

[CR21] Gibson-Graham JK (2014). Rethinking the economy with thick description and weak theory. Current Anthropology.

[CR22] Greene S, Hogan Diane (2005). Researching children’s experience: Methods and approaches.

[CR23] Greenspan I, Walk M, Handy F (2018). Immigrant integration through volunteering: The importance of contextual factors. Journal of Social Policy.

[CR24] Grönlund H (2011). Identity and volunteering intertwined: Reflections on the values of young adults. VOLUNTAS: International journal of voluntary and nonprofit organizations.

[CR25] Guibernau MM (2013). Belonging: Solidarity and division in modern societies.

[CR26] Guo S (2014). Immigrants as active citizens: Exploring the volunteering experience of Chinese immigrants in Vancouver. Globalisation, Societies and Education.

[CR27] Habib S, Ward M (2019). Identities, youth and belonging: International perspectives.

[CR28] Hall SM (2015). Super-diverse street: A ‘trans-ethnography’ across migrant localities. Ethnic and Racial Studies.

[CR29] Handy F, Greenspan I (2009). Immigrant volunteering: A stepping stone to integration?. Nonprofit and Voluntary Sector Quarterly.

[CR30] Hoekstra M (2018). Governing difference in the city: Urban imaginaries and the policy practice of migrant incorporation. Territory, Politics, Governance.

[CR31] Hustinx L, Cnaan RA, Handy F (2010). Navigating theories of volunteering: A hybrid map for a complex phenomenon. Journal for the Theory of Social Behaviour.

[CR32] Ishizawa H (2015). Civic participation through volunteerism among youth across immigrant generations. Sociological Perspectives.

[CR33] Joppke C (2007). Beyond national models: Civic integration policies for immigrants in Western Europe. West European Politics.

[CR34] Koefoed L, Simonsen K (2011). ‘The stranger’, the city and the Nation: On the possibilities of identification and belonging. European Urban and Regional Studies.

[CR35] McLean SL, Schultz DA, Steger MB (2002). Social capital: Critical perspectives on community and ‘bowling alone’.

[CR36] Nowell LS, Norris JM, White DE, Moules NJ (2017). Thematic analysis: Striving to meet the trustworthiness criteria. International Journal of Qualitative Methods.

[CR37] Putnam RD (2000). Bowling alone: The collapse and revival of american community.

[CR38] Rosenthal G, Bogner A (2009). Ethnicity, belonging and biography: Ethnographical and biographical perspectives.

[CR39] Simsek-Caglar A, Glick Schiller N (2018). Migrants and city-making: Multiscalar perspectives on dispossession.

[CR40] Soja EW (2000). Postmetropolis critical studies of cities and regions.

[CR41] Stake RE (2013). Multiple case study analysis.

[CR43] Stewart K (2008). Weak theory in an unfinished world. Journal of Folklore Research.

[CR44] Taylor-Collins E, Harrison T, Thoma SJ, Moller F (2019). A habit of social action: understanding the factors associated with adolescents who have made a habit of helping others. VOLUNTAS: International Journal of Voluntary and Nonprofit Organizations.

[CR45] Te Riele K, Halse C (2018). Reflecting on belonging, space, and marginalised young people. Interrogating belonging for young people in schools.

[CR46] Tomlinson F (2010). Marking difference and negotiating belonging: Refugee women, volunteering and employment. Gender, Work & Organization.

[CR47] Vacchelli E, Peyrefitte M (2018). From a/topia to topia: Towards a gendered right to the city for migrant volunteers in London. Cities.

[CR48] Vertovec S (2007). Super-diversity and its implications. Ethnic and Racial Studies.

[CR49] Vertovec S (2019). Talking around super-diversity. Ethnic and Racial Studies.

[CR50] von Essen J, Askeland H, Espedal G, Jelstad Løvaas B, Sirris S (2020). The art of making sense of volunteering. Understanding values work.

[CR51] Wessendorf S (2014). Commonplace diversity: Social relations in a super-diverse context.

[CR52] Wiertz D (2016). Segregation in civic life: Ethnic sorting and mixing across voluntary associations. American Sociological Review.

[CR53] Wilson J (2012). Volunteerism research: A review essay. Nonprofit and Voluntary Sector Quarterly.

[CR54] Wimmer A, Glick Schiller N (2003). Methodological Nationalism, the social sciences, and the study of migration: An essay in historical epistemology. International Migration Review.

[CR55] Wright S (2015). More-than-human, emergent belongings: A weak theory approach. Progress in Human Geography.

[CR56] Youkhana E (2015). A conceptual shift in studies of belonging and the politics of belonging. Social Inclusion.

[CR57] Yuval-Davis N (2006). Belonging and the politics of belonging. Patterns of Prejudice.

